# Low Intensity Pulsed Ultrasound for Bone Tissue Engineering

**DOI:** 10.3390/mi12121488

**Published:** 2021-11-30

**Authors:** Colleen McCarthy, Gulden Camci-Unal

**Affiliations:** 1 Department of Chemical Engineering, University of Massachusetts Lowell, One University Avenue, Lowell, MA 01854, USA; cmmccar@bu.edu; 2 Department of Surgery, University of Massachusetts Medical School, 55 Lake Avenue North, Worcester, MA 01605, USA

**Keywords:** LIPUS, mechanotransduction, mechanotherapy, bone tissue engineering, 3D scaffolds

## Abstract

As explained by Wolff’s law and the mechanostat hypothesis, mechanical stimulation can be used to promote bone formation. Low intensity pulsed ultrasound (LIPUS) is a source of mechanical stimulation that can activate the integrin/phosphatidylinositol 3-OH kinase/Akt pathway and upregulate osteogenic proteins through the production of cyclooxygenase-2 (COX-2) and prostaglandin E_2_ (PGE_2_). This paper analyzes the results of in vitro and in vivo studies that have evaluated the effects of LIPUS on cell behavior within three-dimensional (3D) titanium, ceramic, and hydrogel scaffolds. We focus specifically on cell morphology and attachment, cell proliferation and viability, osteogenic differentiation, mineralization, bone volume, and osseointegration. As shown by upregulated levels of alkaline phosphatase and osteocalcin, increased mineral deposition, improved cell ingrowth, greater scaffold pore occupancy by bone tissue, and superior vascularization, LIPUS generally has a positive effect and promotes bone formation within engineered scaffolds. Additionally, LIPUS can have synergistic effects by producing the piezoelectric effect and enhancing the benefits of 3D hydrogel encapsulation, growth factor delivery, and scaffold modification. Additional research should be conducted to optimize the ultrasound parameters and evaluate the effects of LIPUS with other types of scaffold materials and cell types.

## 1. Introduction

Approximately 7.9 million bone fractures occur each year in the United States, and 5–10% of these fractures are non-union fractures or have delayed healing times [1]. The complications associated with non-union or delayed-union fractures prolong patient discomfort and immobility and require expensive medical treatments. As a result of these complications, over two million bone transplants are performed each year, making bone the second most transplanted tissue [2]. While bone grafts can be a successful treatment option, bone donors are not widely available, and transplants are associated with high rates of infection or disease transmission [3,4,5]. In response to these problems, bone tissue engineering techniques which use biomaterial scaffolds seeded with stem cells and/or biological molecules, have been developed as a substitute for traditional bone grafts. Alternatively, low intensity pulsed ultrasound (LIPUS) has been shown to promote osteogenesis by activating kinase pathways and upregulating the translation of osteogenic proteins. This paper will explore the theory and biological mechanisms behind the mechanical stimulation of bone and review current literature to evaluate how LIPUS is effective at improving bone tissue formation within 3D scaffolds. The use of LIPUS with various scaffold materials including titanium, ceramics, and hydrogels will be analyzed in vitro and in vivo. Additionally, the paper will address devices that can be used to produce LIPUS and the ultrasound parameters that are most effective for bone tissue engineering purposes.

## 2. Mechanisms of Bone Healing and Mechanical Stimulation

### 2.1. Bone Structure, Bone Remodeling, and Osteogenesis

Bone is an active and dynamic tissue that is constantly changing in response to chemical and mechanical stimuli. The main functions of bone are to provide mechanical support, produce blood cells, regulate metabolic activity by secreting hormones, and balance the pH and ion concentrations in blood [6]. The two main types of bone tissue are cortical bone and trabecular bone. Cortical bone, which makes up approximately 80% of the total bone mass in the body, is comprised of layers of collagen fibrils [7,8]. Alternatively, trabecular bone has a porous structure. Cortical bone primarily provides mechanical strength while trabecular bone plays an important role in regulating metabolic activities [7].

Most bone tissue is formed by a process known as endochondral ossification. In this process, mesenchymal stem cells first differentiate into chondrocytes, which are cartilage cells. As the cartilage cells proliferate, they secrete extracellular matrix proteins. The chondrocytes then undergo hypertropia and apoptosis. In the next phase, osteoclast cells degrade tissue in the center of the cartilage matrix, allowing blood vessels to form. Additionally, osteoblast cells attach to the existing cartilage matrix and secrete bone matrix. In this process, osteoblasts use the model that was created by the chondrocyte cells to build bone tissue [9]. Bone fracture healing also occurs by endochondral ossification that is initiated by the formation of a hematoma at the injury site [6]. In cases where chondrocytes are unable to provide a natural support structure, a synthetic scaffold, made from biomaterials such as calcium phosphates, polyethylene glycol, chitosan, or hydrogels can be supplied [6].

Once bone is formed, it must constantly adapt to its environment to effectively perform its structural and metabolic functions. The process responsible for bone tissue adaptation is called bone remodeling. Bone remodeling is crucial for maintaining the health and strength of bone tissue. Disturbances to the equilibrium between bone formation and bone resorption can lead to diseases such as osteoporosis, rheumatoid arthritis, osteopetrosis, and Paget’s Disease [10].

Bone remodeling primarily depends on the activity of osteoblast and osteoclast cells [11]. Osteoblast cells, which are derived from mesenchymal stem cells, are responsible for forming new bone tissue. Osteoclast cells break down and resorb old or damaged bone tissue. Osteoblast and osteoclast cells communicate with each other via direct contact, cytokines, and extracellular matrix interactions to regulate the formation and degradation of bone tissue [11]. The bone remodeling process consists of four phases: the activation phase, the resorption phase, the reverse phase, and the formation phase.

In the activation phase, bone remodeling is initiated by fractures, changes in mechanical loading, or changes in the chemical environment. Specifically, changes in the concentrations of insulin growth factor- I (IGFI), tumor necrosis factor-α (TNF-α), parathyroid hormone (PTH), and interlueukin-6 (IL-6) activate dormant osteoblast lining cells and initiate the bone remodeling process [12,13]. The activation of lining cells leads to the release of Receptor Activator of Nuclear κB (RANKL), which activates pre-osteoclasts and causes them to fuse together [12,14].

The resorption phase is characterized by the attachment of fused osteoclast cells to the bone surface and the breakdown of bone material. Osteoclast cells break down the organic components of the bone matrix by releasing acidic compounds, such as hydrochloric acid, to dissolve hydroxyapatite [14]. Similarly, osteoclast cells release lysosomal enzymes, such as cathepins K and matrix metallopeptidase 9 (MMP9), to break down the organic portion of bone [12,15]. The breakdown of the bone matrix leaves indents or depressions called Howship Lacunae. The formation of these indents initiates the reverse phase where reverse cells remove debris and release growth factors, such as bone morphogenic proteins (BMP’s), fibroblast growth factors (FGF’s), and transforming growth factors β (TGF β) [10]. These growth factors are needed to recruit osteoblast cells to the area for bone formation.

**Figure 1:**
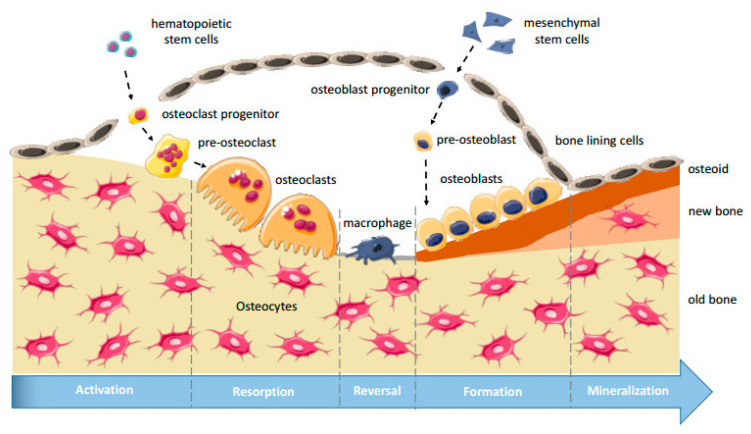
Schematic representation of the bone remodeling process. Bone lining cells are activated by the release of factors such as insulin growth factor- I (IGFI), tumor necrosis factor-α (TNF-α), parathyroid hormone (PTH), and interlueukin-6 (IL-6). Osteoclast cells then break down the organic and inorganic portions of old bone tissue, forming indents. Osteoblasts fill these indents and release a matrix which is then mineralized to form bone tissue. Reprinted with permission under a Creative Commons Attribution 4.0 International license from the Reference by Truesdell et al. [17]. Copyright 2020, AIMS Press.

The formation phase includes the attachment of osteoblast cells to the Howship Lacunae. Osteoblasts are responsible for building the new bone matrix by secreting type I collagen and other non-collagenous proteins. Additionally, osteoblasts play a role in bone mineralization [16]. Bone remodeling can be monitored by measuring the levels of different chemical markers. Bone formation typically results in an increase in alkaline phosphatase (ALP) and osteocalcin (OCN) [10]. Figure 1 displays a schematic representation of the bone remodeling process.

### 2.2. Mechanotransduction and Biological Mechanisms of LIPUS

Changes to bone tissue are regulated by mechanical loading, chemical/hormonal signals, and damage to bone tissue. The effects of mechanical loading are of significant interest to this paper because ultrasound can be used as a source of mechanical stimulation [18]. As described by Wolff’s law, the architecture and strength of bone is affected by the amount of mechanical stress applied to the bone [19]. Increases in mechanical loading cause the cortical bone to strengthen, and decreases in mechanical loading cause the cortical bone to weaken [10].

One way to mechanically stimulate bone tissue is through the propagation of low intensity pulsed ultrasound (LIPUS) waves. The mechanism by which mechanical stimulation affects bone tissue is not completely understood, and several studies have reported that a greater understanding of the mechanism of LIPUS is needed [20,21,22,23]. An earlier study completed by Azuma et al. (2001) showed that LIPUS increased the rate of fracture healing when it was applied during any stage of the bone healing process [24]. The ability for LIPUS to impact many different phases of bone healing suggests that there are many different mechanisms through which LIPUS acts [25].

Before mechanical loading can have an impact on bone, the mechanical signal must be converted to a biochemical signal via a process called mechanotransduction. Mechanotransduction can be broken into four different phases: mechanical coupling, biochemical coupling, signal transmission, and effector cell response [26]. Currently, there is significant evidence showing that osteocyte cells play an important role in sensing and amplifying mechanical signals experienced by bone tissue in the body [27,28]. Osteocytes are the most common type of bone cell and have a stellate or dendritic shape with cytoplasmic processes extending out from the center of the cell. Osteocytes are surrounded by fluid and are located in spaces in the extracellular matrix (ECM) called lacunae. The cytoplasmic processes extend into the canaliculi, which are channels in the ECM [26]. Gap junctions exist between the tips of the cytoplasmic processes allowing the osteocyte cells to form a 3D network through which signaling molecules and nutrients can be passed [29]. In addition to communicating with each other, osteocytes communicate with osteoblast and osteoclast cells to regulate bone remodeling [30].

A mechanical load causes the fluid surrounding osteocyte cells to flow. The movement of fluid exerts shear stress on the osteocytes [31]. To elicit a biological response, these forces must be sensed by receptors known as mechanosensors. The majority of research on mechanosensors for LIPUS has focused on integrin proteins and the activation of kinase pathways [32]. The activation of integrins causes them to bind together to form structures called focal adhesions or focal contacts [33]. Focal adhesions provide a link between the cytoskeleton and the extracellular matrix and are important for the adhesion of cells to the ECM [32].

Focal adhesions contain many types of proteins including focal adhesion kinase (FAK), talin, vinculin, paxillin, and p130Cas [26]. Studies have shown that LIPUS can phosphorylate FAK [34,35]. In response to the activation of FAK, phosphor-inositol 3 kinase (PI3K) and protein kinase B (AKT) are also phosphorylated, which activates the integrin/phosphatidylinositol 3-OH kinase/Akt pathway [34,35]. Additionally, extracellular signal-regulated kinase (ERK), which is required for gene transcription and related to cell proliferation, survival, gene regulation, and cell migration, has also been shown to be activated in response to ultrasound stimulation [33,36]. Whitney et al. (2012) suggest that ERK activation occurs through the MAPK/ERK pathway [36]. Carina et al. (2017) confirmed the effect of LIPUS on the MAPK/ERK pathway by showing that LIPUS increased the expression of MAPK1 and MAPK6 [37]. Both kinase pathways mentioned above have been shown to upregulate runt-related transcription factor 2 (RUNX2) and osterix (OSX), which are both osteogenic transcription factors [26].

**Figure 2:**
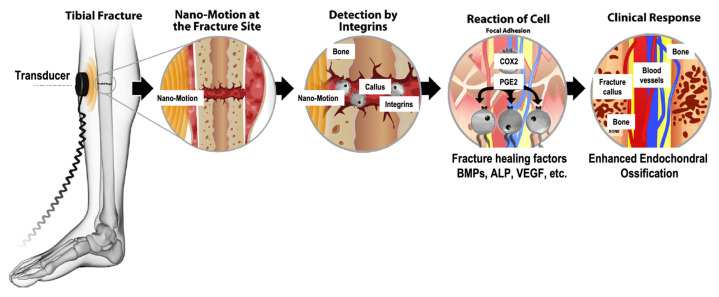
The mechanism of LIPUS on bone tissue repair. The LIPUS waves from the transducer induce forces that activate integrins located in the cell membrane. The activation of integrins leads to the formation of focal adhesions, the phosphorylation of FAK, and the activation of the integrin/phosphatidylinositol 3-OH kinase/Akt pathway. This pathway leads to the formation of COX-2 and PGE2, which are critical for bone formation and fracture healing. Modified from the open access article by Harrison et al. [38]. Copyright 2016, Elsevier Ltd.

By using various inhibitors, Tang et al. (2006) showed that the integrin/phosphatidylinositol 3-OH kinase/AKT pathway led to the formation of cyclooxygenase-2 (COX-2) [34]. COX-2 is one of the key enzymes required to produce prostaglandin E2 (PGE2), which plays a significant role in bone formation by increasing mineralization and upregulating the expression of osteogenic proteins, such as RANKL and BMP [24,34,38]. Additionally, COX-2 leads to the upregulation of osteocalcin, bone sialoprotein, and insulin growth factor [39]. By using NSAIDs as COX-2 inhibitors, studies have concluded that COX-2 inhibition prevented fracture healing [40]. Additionally, inhibition of COX-2 with NSAIDs negatively affects the osteogenic differentiation of mesenchymal stem cells [41]. Furthermore, the inhibition of COX-2 with NS-398 reduced the positive of effects of LIPUS on mineralization [34]. Figure 2 schematically displays the mechanotransduction of ultrasound waves into a biological response.

In addition to activating signaling pathways and osteogenic gene transcription, LIPUS can improve vascularization. Studies have demonstrated that LIPUS increases the expression of vascular endothelial growth factor (VEGF) [37,42]. Similarly, LIPUS has been shown to upregulate IL8, which is an important signaling molecule in the angiogenesis pathway [37]. Furthermore, LIPUS can promote tube formation in human umbilical vein endothelial cells [43] and increase blood vessel number [44] and size [45].

Additional mechanotransduction mechanisms have been proposed including the activation of calcium ion channels/calcium signaling, cilia activation, and β-catenin signaling via the Wnt signaling pathway [26]. While there is evidence for these mechanisms with other forms of mechanical stimulation, such as vibrations, shockwaves, and electrical stimulation, the effect of LIPUS on these mechanisms has not been widely studied [26]. There is evidence, however, that LIPUS can improve the formation of gap junctions, which are important for intercellular communication and signal transmission [46]. Additional research is needed to confirm the exact mechanotransduction mechanism for LIPUS signals. Some insight into the biological mechanism of LIPUS can be explored by evaluating the effects of cell type specific gene deletions on the response of cells to mechanical loading. Table 1 summarizes the results of several cell type specific gene knockout experiments that evaluated how specific genes impact mechanotransduction.

### 2.3. The Mechanostat Hypothesis

The magnitude of a mechanical load and the degree of induced deformation dictates the type of biological response that occurs. Harold Frost proposed the mechanostat hypothesis, which defined four different ranges of strain [65]. He also described the biological response that occurs for strains that fall within each range. Strain is the ratio of the change in the length of a material to its original length. Strain is typically expressed as a decimal, percentage, or in units of µstrain, which is defined as 1 µm/m.

The four strain ranges defined by Frost include the disuse range, the physiological range, the overuse range, and the pathological overuse (fracture) range [66]. Strains below 50–100 µstrain fall into the disuse range and result in higher rates of bone resorption and a decrease in bone mass. Strains between 100 and 1000 µstrain are classified as the physiological range. This type of strain results in microfractures, but it does not have a significant impact on bone mass. The overuse range is defined by strains between 1000 and 3000 µstrain. Strains of this magnitude induce microfractures, increase the rate of bone formation, and increase bone mass. Strains above 3000 µstrain are classified as the pathological overuse range because they can lead to stress fractures or compromise the integrity of bone tissue. When strains are greater than 25,000 µstrain, macroscopic fractures can occur [66].

In addition to identifying the significance of strain, Frost also identified other variables that influence bone mass. These variables include the frequency of mechanical loading, the cycle time, and the amount of time between loading events [66]. The frequency of a strain determines whether a bone cell can restore its original shape after deformation occurs. If the frequency of a mechanical load is too high, bone cells are not able to recover and cannot produce an appropriate response to a load [66].

It should be noted that Frost’s mechanostat hypothesis was developed from experiments on bone tissue. The required amount of strain needed to induce osteogenesis in bone tissue differs from the strain needed for cells in vitro [66]. Bone cells typically respond to mechanical loads when the strain is in the range of 10,000–100,000 µstrain [66]. This amount of strain is 10–100 times greater than the strain needed for bone tissue. It is suspected that skeletal bone requires lower levels of strain to elicit a biological response because the structure of bone tissue allows for strain amplification [66].

In an attempt to explain why bone cells require higher levels of deformation compared to bone tissue, You et al. (2000) completed a study to determine whether direct deformation of bone cells or deformation caused by fluid flow was more significant for inducing osteogenic responses [67]. The research team measured the levels of cytosolic calcium and osteopontin expression for cells subjected to direct substrate deformation or fluid flow. They concluded that direct substrate strain had to be above 10% (100,000 µstrain) to significantly increase cytosolic calcium mobilization. Direct strain levels below 0.5% (5000 µstrain), which is the magnitude of strain that would accompany everyday activities, was not able to increase cytosolic calcium mobilization [67]. These results were supported by examining the response of cells subjected to strain caused by fluid flow. When a fluid induced a wall shear stress of 2 N/m^2^, which is similar to the magnitude of shear stress induced by normal activities, the cells showed significant increases in cytosolic calcium and osteopontin expression [67]. It was concluded that the forces caused by fluid flow are more significant for cell stimulation than direct substrate deformation [67]. These results help to explain why greater levels of strain are needed for in vitro experiments.

### 2.4. Mechanotherapy

Mechanotherapy is defined as the use of mechanical forces to induce tissue healing or cure a disease. The mechanical forces used for mechanotherapy can originate from the movement or physical activity of the patient, or they can originate from an external source. One type of mechanotherapy includes acoustic therapy, which utilizes sound waves to transfer a mechanical force to a biological tissue. Currently, three different types of acoustic therapy have been used for osteogenic purposes. These therapies include low intensity pulsed ultrasound (LIPUS), extracorporeal shock therapy (ESWT), and radial pressure wave therapy (RPWT) [19]. This paper focuses specifically on LIPUS, which is the most common type of acoustic therapy used in clinical settings for bone healing.

## 3. Low Intensity Pulsed Ultrasound (LIPUS)

Ultrasound waves are sound waves that have a frequency above 20 kHz, which is the top end of the human audible range. Specifically, low intensity ultrasound is defined as ultrasound waves that have a spatial average temporal average intensity (ISATA) below 150 mW/cm^2^ [18]. Intensity is a measure of the energy transferred by a sound wave and the rate at which the energy is transferred. In a study completed by Harle et al. (2001), the osteogenic response of in vitro cells was determined to be a function of the ultrasound intensity used for stimulation [68]. The amount of energy transferred to bone affects both the temperature and strain of the tissue.

The effect of intensity on the biological response of cells is not independent and can be influenced by the frequency of the ultrasound wave. For example, a strain of 1 µstrain at a frequency of 1.5 MHz produces the same response as a strain of 10,000 µstrain at a frequency of 1 Hz [19]. Studies have been completed to determine the optimal intensity and frequency settings for inducing osteogenic responses. A summary of the results of these studies is presented in Table 2 and will be discussed later in the paper.

Due to its low intensity, the effects of LIPUS are presumed to be mostly non-thermal. LIPUS, however, can induce small temperature changes in tissue. Since much of the bone remodeling process relies on the activity of enzymes, temperature changes can have a significant effect on the formation of bone. Bone is a dense tissue that has a high attenuation coefficient compared to other types of biological tissues. As a result, a significant portion of the energy transferred to bone by ultrasound waves is lost as heat [69]. In a study by Chang et al. (2002), low intensity ultrasound was found to cause temperature changes of less than 1 °C [70]. Similarly, Duarte et al. (1983), who was the first to develop and use low intensity pulsed ultrasound with bone tissue, recorded temperature variations of 0.01 ± 0.005 °C [71]. High intensity ultrasound can cause much larger temperature changes and lead to tissue damage. Specifically, ultrasound intensities within the range of 5000–25,000 mW can lead to necrosis and further delay fracture healing [19].

## 4. LIPUS Devices

In 1994, the FDA approved LIPUS for use with fracture healing. The FDA has approved several devices that are categorized as bone growth stimulators. The most popular non-implantable, ultrasound device is the Exogen Ultrasound Bone Growth Stimulator developed by Bioventus (Durham, NC, USA). This device produces low intensity pulsed ultrasound (LIPUS) waves whereas other bone growth stimulators provide mechanical stimulation through the development of electrical or electromagnetic fields [1]. The clinically approved ultrasound waves produced by the Exogen Ultrasound Bone Growth Stimulator have an ISATA of 30 mW/cm^2^, a frequency of 1.5 MHz, a pulse repetition rate of 1 kHz, and a pulse width of 200 µseconds. The radiating area of the wave is 3.88 cm^2^, and the temporal average power is 177 mW.

Many in vitro and in vivo studies use ultrasound waves with the clinically approved parameters. Some studies, however, have used other wave intensities or frequencies in their experiments. No consensus has been made on which LIPUS parameters are optimal for promoting bone formation via tissue engineering techniques. Additional studies on this question are required. An analysis of the current research on optimal LIPUS parameters for bone tissue engineering will be discussed later in the paper.

Bone growth stimulators are class III medical devices and are highly regulated by the FDA. Additionally, they are very expensive and cost several thousand dollars. Several studies have used the FDA approved Exogen Bone Growth Stimulator to produce ultrasound waves for their experiments [72,73,74]. Many other studies opted to use the Sonicator 740, which can produce ultrasound waves with a variety of wave parameters [21,23,75,76,77]. Some lab groups have also built their own systems using a waveform generator coupled with an ultrasound transducer [18,78].

## 5. Applications of LIPUS for Bone Tissue Engineering

The use of LIPUS within the field of bone tissue engineering has been addressed by various research teams. In this paper, we reviewed the relevant studies which examined cell morphology and attachment, cell proliferation and viability, osteogenic differentiation, mineralization, bone volume, vascularization, and osseointegration. Additionally, the effect of LIPUS on the biomechanics of scaffolds was explored. When available, the results from both in vitro and in vivo experiments were discussed for each characteristic listed above. The literature studies used various types of stem cells including mesenchymal stem cells (MSCs), MC3T3-E1 pre-osteoblast cells, and dental follicle cells (DFCs). Additionally, various types of scaffold materials were used including ceramics, titanium, and hydrogels. Table 2 displays a summary of the results of relevant studies using LIPUS for bone tissue engineering in 3D scaffolds.

**Table 2:** Summary of relevant studies and results for the use of LIPUS to improve 3D bone engineering techniques.

Study	Cell and Scaffold Type	Ultrasound Parameters	Findings
Veronick et al. (2016) [18]	Cell Type: MC3T3 mouse osteoblast cells Scaffold Material: type 1 collagen hydrogels	Frequency: 1 MHz wave with 1 kHz repetition frequency Pulse mode: 20, 50, or 100% duty cycle Intensity: 30 mW/cm^2^	LIPUS produced a measurable force and hydrogel deformation. LIPUS increased alkaline phosphatase and osteocalcin gene expression. The effect on gene expression was indirectly proportional to hydrogel stiffness and directly proportional to duty cycle.
Zhou et al. (2016) [20]	Cell Type: human mesenchymal cells (hMSCs) Scaffold Material: polyethylene glycol diacrylate bio inks containing RGDS or nHA	Intensity: 150 mW/cm^2^ Frequency: 1.5 MHz Duty cycle: 20%	LIPUS increased MSC proliferation, alkaline phosphatase activity, mineralization, and total protein content in a 3D printed RGDS nHA scaffold.
Feng et al. (2019) [21]	Cell type: MC3T3-E1 mouse pre-osteoblast cells Scaffold Material: Ti6Al4V	Intensity: 40 mW/cm^2^ Pulse Length: 1 ms Frequency: 1 MHz and 3.2 MHz Exposure: 20 min daily for either 3 weeks or 6 weeks.	LIPUS had no significant impact on cell proliferation, increased alkaline phosphatase activity and osteocalcin expression, and increased volume and amount of new bone formation No significant difference was found between 1 MHz and 3.2 MHz frequencies. The 1 MHz frequency was slightly better for ALP activity, OCN content, scaffold pore occupancy, bone area percentage, and calcium deposition, but the difference was not statistically significant.
Kuang et al. (2019) [22]	Cell Type: dental follicle cells (DFCs) Scaffold Material: OsteoBone^TM^ ceramic	Intensity: 90 mW/cm^2^ Frequency: 1.5 MHz Pulse Repetition: 1 kHz Pulse Duration: 200 μs Exposure: 20 min daily for 3, 5, 7, 9, or 21 days	In vitro, LIPUS increased ALP, Runx2, OSX, and COL-I gene expression and the formation of mineralized nodules. In vivo, LIPUS treatment improved fibrous tissue and blood vessel growth.
Wu et al. (2015) [23]	Cell Type: MC3T3-E1 mouse pre-osteoblast cells Scaffold Material: silicon carbide (SiC)	Intensity: 30 mW/cm^2^ Frequency: 1 MHz Pulse length: 1 ms Pulse repetition: 100 Hz Exposure: 20 min for 4 or 7 days	LIPUS improved cell density, cell ingrowth, dsDNA content, and alkaline phosphatase activity
Carina et al. (2017) [37]	Cell Type: human mesenchymal stem cells (hMSCs) Scaffold Material: magnesium dopped hydroxyapatite and type 1 collagen composite (MgHA/Coll)	Intensity: 20 mW/cm^2^ Frequency: 1.5 MHz Pulse repetition: 1 kHz Burst length: 200 μs Exposure: 20 min per day for 5 d/wk for 1 or 2 weeks	LIPUS improved hMSC viability and upregulated several osteogenic genes (ALPL, BGLAP, MAPK1, MAPK6, and VEGF).
Zhu et al. (2020) [44]	Cell Type: MC3T3-E1 mouse pre-osteoblast cells (for in vitro Alizarin red staining experiments) Scaffold Material: poly-L-lactic acid/polylactic-co-glycolic acid/poly-ε-caprolactone (PLLA/PLGA/PCL)	Intensity: 30 mW/cm^2^ Exposure: 20 min daily for 12 weeks	LIPUS improved load carrying capacity, accelerated bone formation, angiogenesis, and differentiation. LIPUS was used to alleviate the effects of osteonecrosis.
Iwai et al. (2007) [72]	Cell Type: MC3T3-E1 mouse pre-osteoblast cells Scaffold Material: hydroxyapatite	Intensity: 30 mW/cm^2^ Frequency: 1.5 MHz Burst width: 200 μs Wave Repetition: 1 kHz Exposure: not specified	LIPUS did not affect biomechanics/compressive strength of hydroxyapatite ceramic LIPUS improved osteoblast number and bone area in the center of implanted, porous scaffold. LIPUS improved volume of mineralized tissue and MC3T3-E1 migration.
Wang, J et al. (2014) [73]	Cell Type: bone marrow stromal cells (BMSCs) Scaffold Material: β-tricalcium phosphate composite	Frequency: 1.5 MHz Burst width: 200 μs Wave Repetition: 1 kHz Intensity: 30 mW/cm^2^ Exposure: 20 min daily for 5, 10, 25, or 50 days	LIPUS increased ALP activity and OCN content. Additionally, LIPUS improved the degree of soft tissue repair, increased blood flow, and resulted in more extensive bone repair. LIPUS did not impact the compressive strength of the β-TCP scaffold.
Hui et al. (2011) [74]	Cell Type: mesenchymal stem cell derived osteogenic cells Scaffold Material: tricalcium phosphate	Frequency: 1.5 MHz Burst width: 200 μs Wave Repetition: 1 kHz Intensity: 30 mW/cm^2^ Exposure: 20 min daily; 5 d/wk, 7 weeks	LIPUS increased spinal fusion at L5 and L6 in New Zealand white rabbits.
Cao et al. (2017) [75]	Cell Type: MC3T3-E1 pre-osteoblast cells Scaffold Material: Ti6Al4V	Frequency: 1 MHz Pulse length: 1 ms Pulse repetition: 100 Hz Intensity: 30 mW/cm^2^ Exposure: 20 min daily for: 1, 4, or 7 days (in vitro) 3 or 6 weeks (in vivo)	An intensity of 30 mW/cm^2^ was found to be most effective at promoting osteogenic differentiation In vitro: LIPUS had no effect on cell proliferation but increased ALP activity, OCN content, and cell ingrowth into the scaffold. In vivo: LIPUS increased/improved amount and volume of new bone formed and the bone maturity.
Liu et al. (2020) [76]	Cell Type: bone marrow stromal cells Scaffold Material: Ti6Al4V coated with BaTiO_3_	Frequency: 1.5 MHz sine wave repeating at 1 kHz Pulse duration: 200 μs Intensity: 30 mW/cm^2^ Exposure: 10 min daily for 7 or 14 days	When combined with BaTiO_3_ LIPUS increased ALP activity and expression of Runx-2, Col-1, and OPN on a titanium scaffold. LIPUS improved the amount of new bone formed (greater volume and filled the scaffold pores to a greater degree).
Fan et al. (2020) [77]	Cell Type: bone marrow mesenchymal stem cells Scaffold Material: Ti6Al4V with BaTiO_3_ coating	Intensity: 30 mW/cm^2^ Frequency: 1.5 MHz Pulse Repetition: 1 kHz Pulse duration: 200 μs Exposure: 10 min daily for 4, 7, or 14 days	In vitro: LIPUS improved cell adhesion, proliferation, and gene expression on a titanium scaffold especially when paired with BaTiO_3_ coating to induce the piezoelectric effect. In vivo: LIPUS improved new bone formation, osteointegration, mineral apposition rate (MAR), and bonding strength of bone and scaffold.
Veronick et al. (2018) [78]	Cell Type: MC3T3-E1 mouse pre-osteoblast cells Scaffold Material: type 1 collagen hydrogels	Frequency: 1 MHz wave with 1 kHz repetition frequency Pulse mode: 20, 50, or 100% duty cycle Intensity: 30 mW/cm^2^	Hydrogel deformation was a function of hydrogel stiffness and duty cycle. LIPUS upregulated COX-2 and PGE_2_ expression. Effects of LIPUS and hydrogel encapsulation were additive.
Wang, Y et al. (2014) [79]	Cell Type: human bone marrow derived mesenchymal stem cells (hMSCs) Scaffold Material: RGD grafted oxidized sodium alginate/N-succinyl chitosan hydrogel (RGD-OSA/NSC)	Duty Cycle: 20% Frequency: 1 MHz Intensity: 200 mW/cm^2^ Exposure: 10 min daily for 1, 3, 7,10, 14, 0r 21 days	LIPUS improved cell proliferation, ALP activity, and mineralization.
Hsu et al. (2011) [80]	Cell Type: MG63 osteoblast-like cells Scaffold Material: commercial purity titanium (CP-Ti)	Intensity: 0, 50, 150, and 300 mW/cm^2^ Frequency: 1 MHz Pulse Repetition: 100 Hz Exposure: 3 min daily for 5 days (in vitro); 10 min daily for 20 or 30 days (in vivo)	LIPUS improved cell viability and ALP activity in vitro. LIPUS improved blood flow and the maturation of collagen fibers. Pulsed ultrasound was better than continuous ultrasound for
Nagasaki et al. (2015) [81]	Cell Type: adipose derived stem cells (ADSCs) Scaffold Material: nanohydroxyapatite (nHA)	Intensity: 60 mW/cm^2^ Frequency: 3.0 MHz sine waves repeated at 100 Hz Exposure: 10 min daily for 7, 14, or 21 days	LIPUS increased calcium and phosphate deposition and bone thickness for adipose derived stem cells in a nHA scaffold.

### 5.1. Cell Morphology and Attachment

Low intensity pulsed ultrasound can change the morphology and attachment of cells seeded on three dimensional scaffolds. In a study by Fan et al. (2020), the morphology and attachment of bone marrow mesenchymal stem cells (BMSCs) on a porous titanium alloy (Ti6Al4V) scaffold was analyzed [78]. The study found that the ratio of total cell area to nucleus area (CN ratio) was significantly increased in the LIPUS groups compared to the control groups after seven days. Additionally, the LIPUS groups had better cell adhesion at day seven as measured by higher vinculin expression. An analysis of SEM images taken on day four revealed that the cells treated with LIPUS had a more spread appearance, a higher density, and cytoplasmic extrusions. The cells in the control group had a flat appearance. In contrast, Cao et al. (2017) found no significant difference between the SEM images for cells treated with LIPUS compared to control groups [75]. In both groups, the cells had long spindle/flat polygon shapes with pseudopodia extending into the scaffold pores.

### 5.2. Cell Viability and Proliferation

LIPUS has proven to be effective for enhancing cell proliferation and viability within 3D bone engineering scaffolds. By using double stranded DNA (dsDNA) content as a measure of proliferation for MC3T3-E1 cells on a silicon carbide scaffold, Wu et al. (2015) found that the dsDNA content was 9% and 27% greater in the LIPUS groups compared to the controls at days four and seven, respectively [23]. Similarly, Wang et al. (2014) found that LIPUS increased the optical density of an MTT assay with human mesenchymal stem cells on days seven, 10, 14, and 21 [79]. Fan et al. (2020) used a Cell Counting Kit-8 (CCK-8) to evaluate cell proliferation of BMSCs on a Ti6Al4V scaffold [77]. The research group found that proliferation was significantly increased on day four and day seven of LIPUS treatment. Furthermore, cell density experiments completed by Zhou et al. (2016) showed that LIPUS increased the density of hMSCs by 5.6–8% on different types of 3D printed scaffolds [20]. There have been some studies where LIPUS was found to have no significant effect on cell proliferation [18,21,32].

The effects of LIPUS on cell viability has also been widely studied. In particular, when studying hMSCs on magnesium-hydroxyapatite/collagen composite scaffolds, Carina et al. (2017) observed a 1.7-fold increase in dsDNA content after 14 days of LIPUS treatment followed by seven days of no LIPUS treatment [37]. Additionally, Fan et al. (2020) showed that LIPUS can significantly improve cell viability after four and seven days [77]. By performing a live/dead assay with BMSCs on titanium scaffolds, cells treated with LIPUS had significantly lower ratios of dead cells to total cells. Additionally, the apoptotic index was 4.9% for non-LIPUS groups compared to 2.8% for LIPUS groups at day four. Statistical analysis concluded that this difference was significant. Similarly, Hsu et al. (2011) determined that LIPUS improved cell viability [80]. By using an MTT assay, the research team found that LIPUS groups had higher levels of metabolic activity compared to control groups. Like cell proliferation, some studies concluded that LIPUS had no significant impact on cell viability [18,75].

### 5.3. Osteogenic Differentiation

Osteogenic differentiation can be evaluated by measuring biochemical markers, such as enzyme activity, gene expression, and protein release, as well as by analyzing the morphology of cells. In the evaluated studies, osteogenic differentiation was most commonly evaluated by measuring the activity and expression of alkaline phosphatase and osteocalcin. The expression and regulation of other osteogenic genes and proteins, including Runx-2, Col-A1, BGLAP, OSX, BMP-2, and TGF-B1, were also analyzed by literature examples.

#### 5.3.1. Early Osteogenic Markers—Alkaline Phosphatase (ALP) Activity

Alkaline phosphatase (ALP) is an early osteogenic marker that aids in calcium deposition [5,20]. ALP is responsible for catalyzing the hydrolysis of pyrophosphate, which regulates the formation of mineral crystals and bone mineralization. The enzymatic activity of ALP typically increases initially as cells differentiate toward osteogenic lineage. The activity then decreases as the extracellular matrix mineralizes [56]. Several of the reviewed studies measured ALP activity, expression, and cytoplasmic release in vivo and in vitro to evaluate the osteogenic differentiation of stem cells exposed to LIPUS.

In a study completed by Carina et al. (2017), which evaluated hMSCs in MgHA/collagen scaffolds, the expression of the ALPL gene, which is responsible for transcribing the ALP enzyme, had a fold of increase of 11.8 after 14 days of LIPUS treatment and 22.0 after 14 days of LIPUS treatment followed by seven days of no LIPUS treatment [37]. In a study by Zhou et al. (2016), LIPUS increased the ALP activity of hMSCs by 4.4–6.6% after two weeks for scaffolds containing nanohydroxyapatite (nHA), Arginine-Glycine-Aspartic acid-Serine (RGDS) cell adhesive peptides, or both nHA and RGDS [20]. At three weeks, ALP activity increased between 5.0–6.8% for LIPUS groups compared to the respective control groups.

A study using collagen hydrogels with varied stiffness levels found significant increases in ALP activity for flexible hydrogels with low collagen concentrations (1 mg/mL) [18]. When the collagen concentration was increased to 2 mg/mL or 3 mg/mL, however, the difference between LIPUS and control groups became non-significant. In this study, Veronick et al. (2016) showed that the flexibility of the hydrogel scaffold and the degree of deformation imposed by LIPUS has a direct effect on the ability of LIPUS to promote osteogenic differentiation [18]. In addition to the studies discussed thus far, several other in vitro studies concluded that LIPUS can significantly increase ALP activity and/or expression between four and 24 days [21,22,23,77,79,80,82].

ALP activity was measured in vivo by Wang et al. (2007) [73]. By implanting β-tricalcium phosphate (β-TCP) scaffolds seeded with BMSCs in seven-week-old male Fischer rats, the research team concluded that LIPUS increased ALP activity on days 5, 10, 25, and 50 compared to a control. It was also noted that ALP activity for all groups was the highest on day 25 compared to all other time points measured.

#### 5.3.2. Late Osteogenic Markers—Osteocalcin (OCN)

In addition to alkaline phosphatase, the expression and release of osteocalcin (OCN) can be used as a marker of osteogenic differentiation. Osteocalcin is considered a late osteogenic marker and is necessary to bind calcium ions and other minerals for bone formation [83]. Cao et al. (2017) found that the osteocalcin content of MC3T3-E1 cells on Ti6Al4V scaffolds increased by 16.2% and 9.5% on days 10 and 14, respectively, when LIPUS was applied [75]. Carina et al. (2017) found similar results when studying hMSCs on an Mg/collagen composite scaffold [37]. The fold of increase of OCN released by cells was greater than five for all time points (seven days of LIPUS treatment, 14 days of LIPUS treatment, and 14 days of LIPUS treatment followed by seven days of no LIPUS stimulation).

Veronick et al. (2016) found that the upregulation of OCN became less prominent as hydrogel stiffness/collagen concentration increased and LIPUS induced less deformation [18]. For a collagen concentration of 1 mg/mL, LIPUS significantly increased osteocalcin expression on day 7. At higher collagen concentrations of 2 mg/mL and 3 mg/mL, the difference between the osteocalcin expression in the LIPUS and control groups was not significant at any time point (1, 3, or 7 days). Additional in vitro studies have shown the positive effects of LIPUS on osteocalcin expression [81,82] and release [21]. Wang et al. (2007) completed an in vivo study and found that the OCN content on β-TCP scaffolds containing BMSCs was significantly higher for groups treated with LIPUS on days 5, 10, 20, and 50 [73]. For all groups, OCN content increased with time.

#### 5.3.3. Other Osteogenic Markers

Osteogenic differentiation can be marked by other biochemical signals, including the expression of cyclooxygenase 2 (COX-2), prostaglandin E2 (PGE2, alpha-1 type-1 collagen (COL-A1), runt related transcription factor 2 (RUNX 2), bone gamma-carboxyglutamate protein (BGLAP), osterix (OSX), and total protein content. Additionally, osteogenic differentiation can be determined by analyzing changes in cell morphology. Veronick et al. (2018) found that LIPUS stimulation at both 30 mW/cm^2^ and 150 mW/cm^2^ resulted in the upregulation of COX-2 and PGE2 in MC3T3-E1 cells encapsulated in collagen hydrogels [78]. Both COX-2 and PGE2 are biomarkers for bone formation that are known to be upregulated in response to fluid forces [84,85].

According to Carina et al. (2017), LIPUS had no significant effect on COL-A1 expression in hMSCs on Mg/collagen composites scaffolds in vitro [37]. In contrast, Kuang et al. (2019) and Zhu et al. (2020) found that COL-A1 expression significantly increased in response to LIPUS in vivo [22,86]. Similarly, Carina et al. (2017) found no significant increase in RUNX2 expression in hMSCs, whereas Kuang et al. (2019) measured a significant upregulation of RUNX2 in DFCs in response to LIPUS [22,37]. While Carina et al. (2017) found no significant upregulation of COL-A1 nor RUNX2, the team measured a 1.58-fold increase for the expression of BGLAP after 14 days of LIPUS treatment [37]. In addition to the genes discussed thus far, other studies have found that the expression of OSX [22] and TGD-B1 [86] were upregulated in LIPUS groups compared to control groups.

Osteogenic differentiation can also be evaluated by measuring the total protein content in cells, as was done by Zhou et al. (2016) [20]. It was concluded that LIPUS increased the total protein content of hMSCs between 14.9–17.3% after two weeks and between 18.6–34.9% after three weeks [20]. Finally, osteogenic differentiation can be analyzed by examining changes in cell morphology. Wang et al. (2014) described that hMSCs encapsulated in RGD-grafted oxidized sodium alginate/N-succinyl chitosan hydrogels showed more prominent osteogenic characteristics, such as spindle shape, after being exposed to LIPUS stimulation for 10 min a day [79]. Based on the results of the various studies discussed, there is overwhelming support for the use of LIPUS for osteogenic induction.

### 5.4. Bone Mineralization

Bone mineralization is the process by which calcium and phosphate minerals, in the form of hydroxyapatite [(Ca)_10_(PO4)_6_(OH)_2_], deposit in the extracellular matrix (ECM) to form a hard tissue that is capable of bearing mechanical loads [87]. For in vitro experiments, mineralization is typically measured using Alizarin red staining, which selectively binds to calcium ions in the bone matrix. After three weeks of culture, Zhou et al. (2016) found that LIPUS increased the amount of calcium deposition by 12.8% and 13.3% for a non-modified and RGDS modified polyethylene glycol diacrylate 3D printed scaffold, respectively [20]. Wang et al. (2014) used both Alizarin red staining and the Calcium C- Test Kit to measure the calcium content in the bone matrix and evaluate bone formation [79]. The results of both tests showed the positive effects of LIPUS on bone mineralization as the number and size of the calcium nodules was significantly greater in the LIPUS groups compared to the controls. In addition to these studies, other in vitro studies also concluded that LIPUS improved mineralization [22,81,82].

LIPUS has also proved to be an effective means of promoting mineralization in vivo. Feng et al. (2019) measured the calcium deposition in Ti6Al4V scaffolds seeded with MC3T3-E1 cells that were implanted into New Zealand white rabbits [21]. The study concluded that LIPUS increased the amount of calcium deposition at weeks three and six. Cao et al. (2017) evaluated bone mineralization by fluorescently staining calcein in porous Ti6Al4V scaffolds implanted into male New Zealand white rabbits [75]. The fluorescent labelling rate, which is the ratio of the fluorescent area to the total area, was significantly higher in LIPUS groups compared to control groups. A higher fluorescence labelling area signifies more active bone formation. Additional studies have also been completed which support the use of LIPUS for improving bone mineralization in 3D scaffolds in vivo [72,77]. In contrast to these in vivo studies, Zhu et al. (2020) found that LIPUS did not significantly increase the calcium nor phosphorus content in ceramic composite scaffolds implanted into rat femoral heads [86].

### 5.5. Bone Area and Volume

One of the major ways of determining whether a scaffold will serve as a successful tool for healing bone defects is by measuring the amount of bone tissue that forms in the scaffold/defect site. Several in vivo studies have been completed to evaluate whether LIPUS can increase the amount of bone tissue formed. Wang et al. (2014) studied BMSCs that were seeded on β-TCP scaffolds implanted in male Fisher rats [79]. The team found that after five days, the rats that received daily LIPUS treatment already showed soft tissue around the scaffolds. No soft tissue appeared in the control group. After 25 days, the LIPUS groups showed greater primary bone formation in the pores of the scaffolds compared to control groups. The LIPUS groups also contained cuboidal cells, which are active osteoblasts. The presence of these cells signified active bone formation.

Seven weeks after implantation between the L5 and L6 vertebrae in New Zealand white rabbits, Hui et al. (2011) observed that LIPUS increased the volume of bone tissue within tricalcium phosphate scaffolds by 32% [74]. Additionally, the distance between the L5 and L6 transverse processes was 67% shorter in LIPUS groups. Furthermore, seven weeks post operation, the fusion rate of the spinal defect in LIPUS groups was 86% compared to 14% in the control groups. Additional studies have found similar results and confirmed that LIPUS can increase the volume [77], area [75], and thickness [81] of bone formed in 3D scaffolds.

Iwai et al. (2007) found that the formation of bone tissue in hydroxyapatite scaffolds implanted in femoral defects in New Zealand white rabbits was the same for LIPUS and control groups at the edges of the scaffold [72]. At the center of the scaffolds, however, the amount of bone tissue was significantly greater in the LIPUS group on week two.

### 5.6. Vascularization and Angiogenesis

Vascularization of newly formed bone tissue is necessary to ensure the bone tissue receives oxygen and nutrients. The formation of blood vessels occurs via three different processes: vasculogenesis, angiogenesis, and arteriogenesis. Vasculogenesis is the formation of new blood vessels from progenitor cells while angiogenesis and arteriogenesis involve the remodeling of an existing vascular network [88]. Vascularization of tissue engineered implants typically occurs spontaneously after it is implanted into the body. This spontaneous vascularization, however, often does not occur fast enough to supply the whole tissue with nutrients. Thus, additional techniques are required to promote vascularization within tissue implants [88]. One possible technique that has been studied for this purpose is LIPUS.

By completing immunohistochemical staining with anti-CD31 antibodies on tissue harvested from β-TCP scaffolds implanted in Fisher rats, Wang et al. (2007) observed that LIPUS treatment for 10 days significantly increased vascularization [73]. Similarly, the expression of CD31 and CD34, which are both endothelial markers, significantly increased for hMSCs in chitosan hydrogels with LIPUS stimulation. Furthermore, Kuang et al. (2019) observed greater vascularization in ceramic scaffolds that were seeded with dental follicle cells (DFCs) as a result of LIPUS treatment [22]. Zhu et al. (2020) observed that LIPUS had no significant impact on the number and diameter of blood vessels that formed in a PLLA/PLGA/PCL scaffold implanted into rats with steroid induced osteonecrosis [86].

### 5.7. Osseointegration

Osseointegration is defined as the growth of bone tissue into an implant material. Most modern-day scaffolds designed for bone tissue engineering are porous. Successful osseointegration involves the growth of tissue into the pores of the scaffold. This growth helps to integrate and stabilize the implant with the surrounding bone. It has been hypothesized that LIPUS can promote osseointegration and improve the success of bone scaffolds for regenerating healthy bone tissue.

One way to evaluate osseointegration is by measuring the amount of bone tissue that occupies the pores and the central areas of the scaffolds. Iwai et al. (2007) showed that bone ingrowth into a porous hydroxyapatite ceramic scaffold occurred more quickly when LIPUS stimulation was applied [72]. Additionally, Iwai et al. (2007) found that the volume of mineralized tissue in the central region of the scaffold was significantly greater in the LIPUS group after two and three weeks of LIPUS stimulation [72]. The number of osteoblast cells found in the central region of the LIPUS treated scaffolds at week 2 was equivalent to the number of osteoblast cells found at week 3 in the control groups. In support of the results, Hui et al. (2011) found that LIPUS resulted in 35% better bony integration of a TCP scaffold implanted into the spines of New Zealand white rabbits [74]. The ratio of the length of osseointegrated tissue to the length of the transverse process was 79.8% for the LIPUS group compared to 54.1% for the control.

Cao et al. (2017) was able to measure the effect of LIPUS on bone ingrowth both in vitro and in vivo [75]. In the in vitro experiments, the cell density was measured at the top, middle, and bottom of a Ti6Al4V scaffold seeded with MC3T3-E1 cells. The density of osteoblast cells in the middle and bottom of the scaffolds was higher for the LIPUS groups compared to the controls. The difference in bone density between LIPUS and the control groups was determined to be statistically significant at the bottom of the scaffold. These results suggest that LIPUS promotes the migration of osteoblast cells into titanium scaffolds. For in vivo experiments, Cao et al. (2017) measured the pore occupancy fraction (POF) of the scaffolds [75]. The volume of new bone in the pores of the scaffold was found to be greater in the LIPUS groups after three and six weeks. Similarly, Huang et al. (2017) found that tibiae defects in rabbits showed greater bone formation and bone ingrowth into a PLLA nanofibrous membrane when LIPUS treatment was applied [89].

In support of these results, Fan et al. (2020) used Van-Gieson staining and observed that titanium scaffolds treated with LIPUS had newly formed bone tissue at the peripheral and central regions [77]. The non-LIPUS groups only contained new bone tissue at the peripheral regions of the scaffold. The LIPUS group was also observed to contain larger pieces of newly formed bone. Fan et al. (2020) also measured osseointegration by measuring the strength of the fusion between the bone and the implant [77]. The peak pull out load, which is the amount of force required to remove a scaffold from the implant site, was significantly greater for the LIPUS groups compared to the controls at six and 12 weeks. This result suggested that LIPUS can strengthen the bond between an implant and the surrounding bone. In addition to these studies, additional research has been completed to show that LIPUS improves osseointegration [21,73].

### 5.8. Scaffold Biomechanics

Since LIPUS induces mechanical stress in materials, it is important to determine whether LIPUS influences the mechanical integrity of scaffolds. The mechanical strength of scaffolds needs to be preserved to achieve the greatest bone regeneration potential [5]. The effect of LIPUS on compressive strength and material deformation has been evaluated for scaffolds made from TCP, hydroxyapatite, and collagen hydrogels. In a study completed by Wang et al. (2014) it was found that LIPUS had no impact on the compressive strength of TCP scaffolds [73]. Similarly, Iwai et al. (2007) observed no change in the compressive strength of hydroxyapatite scaffolds as a result of LIPUS treatment [72].

The impact of LIPUS on the deformation of collagen hydrogel was evaluated in a study completed by Veronick et al. (2016) [18]. By observing and quantifying the movement patterns of fluorescent beads encapsulated in hydrogels, the research team found that deformation occurred only at the onset and offset of ultrasound stimulation. While the ultrasound was kept on, the beads showed little to no movement. The degree of deformation of the hydrogels was also determined to be a function of the collagen concentration (0.05%, 0.075%, 0.1%, and 0.2%) and the duty cycle of the ultrasound (20, 50, or 100%). As the hydrogel stiffness increased and the duty cycle decreased, greater deformation was observed. The results of Veronick et al. (2016) suggest that hydrogel properties as well as LIPUS parameters can be altered to change the amount of force applied to cells [18].

In a later study by Veronick et al. (2018), it was observed that the degree of deformation was greatest at the top of the hydrogel, closest to where the source of the ultrasound was located [78]. Additionally, the hydrogels deformed in the x, y, and z planes. For flexible hydrogels, deformation was primarily observed in the x and y directions, but as stiffness increased, deformation became evenly distributed between the x, y, and z planes. The results clearly demonstrate how mechanical stimulation can alter the shape of hydrogel materials.

## 6. Synergistic Effects of LIPUS with Other Bone Tissue Engineering Techniques

An analysis of the use of LIPUS in bone tissue engineering has shown that LIPUS is effective at improving factors such as osteogenic differentiation, mineralization, volume of newly formed bone, and osseointegration. While LIPUS can be effective on its own, it can be used alongside other bone tissue engineering techniques to further improve bone formation. Specifically, LIPUS has been shown to have synergistic effects by providing the piezoelectric effect. It has also demonstrated additive benefits when combined with bone tissue engineering techniques such as 3D hydrogel encapsulation, BMP-2 delivery, and peptide or mineral modification of scaffolds.

### 6.1. D Encapsulation

Hydrogels are polymeric materials that are frequently used as scaffolds for 3D encapsulation of cells. Hydrogels are composed of cross-linked polymers and can hold large amounts of water by weight. Hydrogels have been successful materials for cell scaffolds because they mimic the natural environment of a cell and provide a three-dimensional scaffold for cell growth [90]. Compared to other types of biomaterials, hydrogels are highly tunable and have been shown to have better biocompatibility, biodegradability, and porosity [91,92,93]. Veronick et al. (2018) showed that LIPUS and hydrogel encapsulation of cells (as opposed to seeding cells on a rigid TCP plate) both upregulated COX-2 and PGE2 expression on their own [78]. When LIPUS and hydrogel encapsulation were used together, however, the expression of COX-2 and PGE2 was greater than when each technique was used on its own.

### 6.2. Piezoelectric Effect

The piezoelectric effect is the ability of a material to produce an electric charge in response to mechanical stress. Bone is a piezoelectric material that produces stress generated potentials as a means of regulating bone growth and mineralization. Thus, the ability for a scaffold to produce electric potentials similar to those produced by bone can improve the scaffold’s ability to mimic the natural environment of bone and promote tissue growth [77]. Barium titanate (BaTiO_3_) is a piezoelectric ceramic that produces an electrical microenvironment similar to the one formed by bone when it is mechanically stimulated [76]. Barium titanate can be used as a coating on titanium alloys to improve bioactivity and the binding of the scaffold to surrounding bone [76].

In a study completed by Liu et al. (2020), the effect of LIPUS on plain Ti6Al4V scaffolds and barium titanate coated Ti6Al4V scaffolds was compared [76]. The in vitro study concluded that BMSC’s seeded on BaTiO_3_ coated scaffolds showed significantly higher ALP activity, RUNX-2 expression, COL-1 expression, and OPN expression after seven and 14 days of LIPUS treatment compared to cells on plain titanium scaffolds.

Liu et al. (2020) also observed the effects of LIPUS in vivo [76]. At four months after scaffold implantation, it was observed that the bone volume in the BiTiO_3_ scaffolds was significantly greater than the bone volume in non-coated scaffolds. Furthermore, the BaTiO_3_ scaffolds also had greater bone volume to total volume ratios (BV/TV) at four and eight months which signified better bone ingrowth. Overall, the studies indicated that piezoelectric materials stimulated with LIPUS can improve osteogenic properties and bone formation in vitro and in vivo.

A similar study on the piezoelectric effect was completed by Fan et al. (2020) where cell attachment, cell proliferation, cell viability, bone volume, and bone ingrowth were measured for BaTiO_3_ coated and non-coated Ti6Al4V scaffolds [77]. The BaTiO3 scaffold proved to be more hydrophobic and have improved surface roughness compared to the non-coated scaffold. The larger surface area on the coated scaffolds caused BMSCs to have better cell attachment and spread over the entire scaffold surface. The cells in the coated scaffolds also had higher cell stretch, higher cell density, and better cytoplasmic extrusions than cells seeded on non-coated titanium scaffolds.

Additionally, Fan et al. (2020) found that cell proliferation increased more significantly when LIPUS and BaTiO_3_ coatings were used together than when either element was used alone [77]. The percentage of dead cells to total cells decreased when LIPUS or BaTiO_3_ was used individually, but it decreased to a greater degree when they were used together. Furthermore, the percentage of newly formed bone and the amount of bone ingrowth in vivo both improved more significantly when LIPUS and BaTiO_3_ were used together compared to when each element was used separately.

Das et al. (2020) investigated the ability of LIPUS to create a surface charge on a piezoelectric nanofiber bone scaffold made from poly(L-lactic acid) (PLLA) [82]. The results demonstrated that PLLA films treated with LIPUS were able to retain a surface charge over a time period of 26 days. As measured by ALP, osteocalcin, and osterix levels in adipose derived stem cells in vitro, the experimental groups for piezoelectric nanoparticles and LIPUS treatment induced significantly greater osteogenic differentiation compared to groups containing non-piezoelectric nanofibers and groups that did not receive LIPUS treatment. These in vitro results were supported by in vivo experiments where mice that received piezoelectric nanofibers with ultrasound treatment displayed greater mineralization, bone formation, ALP release, and osteoblast migration within a critical size calvaria defect.

### 6.3. BMP-2 Delivery

In addition to delivering cells, 3D scaffolds are also frequently used to deliver therapeutic drugs and growth factors, such as bone morphogenic protein 2 (BMP-2). BMP-2 is a growth factor that is known to induce bone growth and osteogenic differentiation [94,95]. In a study completed by Zhu et al. (2020) poly-L-lactic acid/polylactic-co-glycolic acid/poly-ε-caprolactone (PLLA/PLGA/PCL) composite scaffolds were loaded with BMP-2 and implanted into rates with steroid induced osteonecrosis [86]. Bone mineral density, the ratio of bone volume to total volume, trabecular number, and trabecular thickness all improved significantly because of LIPUS treatment. When LIPUS treatment was given in conjunction with BMP-2 delivery, these factors saw a more significant increase.

In addition to the elements already presented, Zhu et al. (2020) found that the carrying capacity and mineralization of newly formed bone improved to a greater degree with both LIPUS treatment and BMP-2 delivery [86]. The maximum bending load of the newly formed bone tissue improved because of LIPUS treatment, but more significant improvements were observed when LIPUS treatment was accompanied by BMP-2 delivery. The same trend was observed for calcium and phosphorus deposition, the number and diameter of blood vessels formed, and the expression of osteogenic proteins such as TGF-B1, RUNX-2, COL-I, and OCN. It was concluded that the synergistic effects of LIPUS and BMP-2 delivery can reduce the side effects of osteonecrosis [86].

Wijdicks et al. (2009) also investigated the ability of LIPUS to enhance BMP induced bone growth [96]. Using collagen sponges with either 1 µg or 5 µg of recombinant BMP-2, the research team found that LIPUS increased bone formation by 117.7 and 2.3-fold, respectively. LIPUS did not influence bone mineral density or total mineral content. The results of the two studies presented strongly support the synergistic effects of using LIPUS treatment with the delivery of BMP-2 to defect sites.

### 6.4. Scaffold Modification with Peptides or Minerals

To improve the properties of biomaterials and render them more bioactive and/or biocompatible, the surfaces of the materials can be modified using combinations of amino acids, such as arginine-glycine- aspartic acid (RGD) or arginine-glycine-aspartic acid-serine (RGDS). Additionally, mineralization with hydroxyapatite allows a scaffold to more closely mimic natural bone and enhances bone regeneration [6,97,98]. The hydrophilic surfaces of hydrogels are not favorable for protein adsorption and cell adhesion and growth. As a result, bone formation and vascularization can be inhibited unless the hydrogel surfaces are modified to make them more hydrophobic. RGD is often used to modify hydrogel surfaces. As shown by Wang et al. (2014), RGD modification of oxidized sodium alginate/N-succinyl chitosan hydrogels combined with LIPUS treatment resulted in the greatest improvements in cell proliferation, endothelial induction, osteogenic induction, and mineralization [79]. Similarly, Zhou et al. (2016) found that cell proliferation, ALP activity, and calcium deposition was greater when the 3D printed scaffolds were modified with RGDS and treated with LIPUS than when only one factor was applied [20].

In addition to RGDS alone, Zhou et al. (2016) also analyzed the impact of LIPUS on scaffolds containing both RGDS and nanocrystalline hydroxyapatite (nHA) [20]. Hydroxyapatite is one of the main components of the bone matrix. It has been shown to improve the bioactivity and biomimicry of biomaterials and improve cell adhesion, proliferation, and osteogenic differentiation [99]. LIPUS had the most profound impact on cell proliferation when it was used with scaffolds modified with both RGDS and nHA. Cell proliferation on RGDS and nHA scaffolds increased by 26.7% after five days of LIPUS treatment. Similarly, the ALP activity and total protein content was the greatest for RGDS and nHA scaffolds that had received LIPUS stimulation. Overall, the study concluded that RGDS, nHA, and LIPUS are effective ways to improve the bioactivity of biomaterials and induce cell proliferation and osteogenic differentiation. The use of these three elements together can lead to even greater improvements in mineralization.

## 7. Optimal LIPUS Parameters for Bone Tissue Engineering

The studies that have been presented in this paper have used a variety of different LIPUS parameters. Since the studies all used different experimental conditions, such as scaffold type, cell type, ultrasound exposure time, and cell culturing techniques, they cannot be directly compared to determine the optimal LIPUS settings. A few studies, however, did compare different LIPUS parameters within their controlled experiments to determine which settings were most appropriate for their experimental conditions.

Feng et al. (2019) completed a study to compare the effects of 1 MHz and 3.2 MHz ultrasound on MC3T3-E1 cells cultured on Ti6Al4V scaffolds in vitro and in vivo [21]. The study concluded that there was no significant difference between the two frequencies for osteogenic differentiation, bone volume and maturity, or scaffold ingrowth/pore occupancy. While the lower frequency (1 MHz) resulted in slightly higher ALP activity, OCN production, and pore occupancy values, the differences between the two frequencies were not large enough to be considered statistically significant.

In addition to frequency, studies have researched the effects of various LIPUS intensities. Cao et al. (2017) determined that a LIPUS intensity of 30 mW/cm^2^, which is same LIPUS intensity clinically approved for fracture healing, increased osteoblast differentiation on Ti6Al4V scaffolds more than intensities of 0, 10, 60, and 100 mW/cm^2^ [75]. An intensity of 30 mW/cm^2^ is further supported by Veronick et al. (2018) who concluded that a 30 mW/cm^2^ ultrasound intensity resulted in higher COX-2 and PGE2 expression than 150 mW/cm^2^ [78]. Similar results were found by Angle et al. (2011) in a 2D study on rat bone marrow stromal cells [100]. The research team concluded that an ultrasound intensity of 30 mW/cm^2^ increased ALP activity by 209% and was more effective than intensities of 2 mW/cm^2^ and 15 mW/cm^2^ at promoting osteoblast differentiation. Despite this finding, the same study found that an intensity of 2 mW/cm^2^ was better at promoting mineralization compared to intensities of 15 and 30 mW/cm^2^. Zhou et al. (2016) used a LIPUS intensity of 150 mW/cm^2^ in their study using hMSCs on 3D printed polyethylene glycol diacrylate scaffolds [20]. This choice was made because an intensity of 150 mW/cm^2^ resulted in better MSC proliferation compared to 20, 50, 75, or 300 mW/cm^2^. It should be noted that the preliminary study completed by this group to determine the ultrasound intensity was done using a 2D culture on a 24 well plate.

Typically, low intensity ultrasound is applied in a pulsed rather than continuous manner. Hsu et al. (2011) compared the performance of pulsed versus continuous ultrasound and determined that pulsed ultrasound was able to increase ALP activity more than continuous ultrasound [80]. Pulsed ultrasound was also more effective than continuous ultrasound at improving cell proliferation after three days of stimulation. The results of this analysis on LIPUS parameters suggests that different ultrasound intensities may be better at promoting different aspects of bone formation. Further studies need to be completed to confirm the most effective ultrasound parameters for osteogenesis overall.

## 8. Limitations and Future Directions of LIPUS

One of the major limitations of LIPUS for bone tissue engineering is the lack of uniformity of experimental studies. Since many different scaffolding materials, LIPUS settings, and treatment times are used by researchers, it is challenging to directly compare study results, complete meta-analyses, or make overall conclusions regarding the impact of LIPUS on bone formation in 3D scaffolds. A second limitation of LIPUS is the lack of consistency in the effectiveness of LIPUS for fracture healing in clinical settings. While many controlled studies have concluded that LIPUS is effective, few others have observed the opposite result [83,101,102,103,104]. LIPUS devices are clinically approved for fracture healing, but more consistent clinical results would be needed to confirm the effectiveness and reliability of LIPUS for bone tissue engineering. Third, ultrasound generation requires costly equipment that can be difficult to obtain. Low intensity ultrasound cannot be produced by most commercial ultrasound generators for medical use. Specialized devices or wave form generators are required for LIPUS generation. Finally, most studies with LIPUS have been completed using metal, ceramic, and hydrogel scaffolds. There is currently a gap in the research of LIPUS with unconventional scaffolding materials, such as paper and plant-based materials, which have been successful for tissue engineering applications [105,106]. The integration of LIPUS with new types of biomaterials is an area where additional research work could be performed.

LIPUS has already been FDA approved for clinical use to treat non-union fractures. As a result, it can be predicted that the use of LIPUS for bone tissue engineering applications could be easily transitioned into the clinic. The next steps in improving the use of LIPUS for bone tissue engineering include determining the optimal LIPUS parameters for specific cell types, defect locations, and scaffold materials. Specifically, it is currently challenging to deliver ultrasound waves of the correct intensity to deep tissues, which limits the potential applications of LIPUS [107]. Finally, ultrasound generators and bone growth stimulators are typically costly, and many medical insurance companies do not cover the costs of these devices. Decreasing the cost of ultrasound equipment can help to promote further research and also increase patient use of LIPUS for bone healing.

## 9. Conclusions

There are several factors that are important for successful bone tissue engineering. These factors include utilizing biocompatible and biodegradable scaffolding materials with sufficient mechanical and structural properties, differentiating stem cells to osteogenic lineage, promoting vascularization in newly-formed tissue, and stimulating tissue growth with growth factors or mechanical stress [5,98,108]. By enhancing osteogenic differentiation, mineralization, osseointegration, and mineralization, LIPUS can help engineers and medical professionals improve the bioactivity, biocompatibility, and integration of bone scaffold materials.

An analysis of in vitro and in vivo studies revealed that LIPUS treatment can result in greater cell proliferation, as measured by cell density and dsDNA content, greater cell metabolic activity, and lower rates of cell death. LIPUS also increased the expression of osteogenic markers, such as ALP, OCN, COX-2, PGE2, and RUNX2, and endothelial markers, such as CD31 and CD34, which signify greater osteogenic differentiation and vascularization, respectively. Furthermore, LIPUS treatment resulted in significantly greater calcium deposition and improved the integration between the scaffold and surrounding bone tissue. LIPUS did not significantly impact the mechanical properties of ceramic scaffolds, and hydrogels showed elastic deformation when exposed to LIPUS.

The reviewed studies also supported that LIPUS has synergistic effects. LIPUS can produce the piezoelectric effect on scaffolds containing piezoelectric materials and has additive effects when used in conjunction with 3D hydrogel encapsulation, BMP-2 delivery, peptide scaffold modification, and mineral scaffold modification. Due to the variety of experimental conditions tested in the reviewed studies, there are currently no universally accepted ultrasound parameters. An intensity of 30 mW/cm^2^ was the most frequently used intensity setting and was shown to be optimal for osteoinduction. Based on a review of various experimental studies, it is suspected that different intensities can be used to optimize different aspects of bone tissue formation including cell proliferation, osteogenic differentiation of stem cells, and mineralization. LIPUS stands as a promising method to mechanically stimulate cells for bone tissue engineering. Additional research should be carried out to determine the optimal ultrasound parameters and gain additional evidence for the positive effects of LIPUS with additional types of scaffolding materials and cell types. Specifically, it would be beneficial to explore the impacts of LIPUS with scaffolds made from unconventional biomaterials, such as silk, paper, and plant-based materials.

## Figures and Tables

**Table 1:** Effects of Cell Type Specific Gene Deletions on the Response of Cells to Mechanical Loading.

Source	Cell Line	Gene Deletion	Effect of Gene Deletion
Arthur et al. (2020) [47]	Osx-Cre	EfnB1	Soft callus and remodeling phases of fracture healing were delayed.
Zhang et al. (2011) [48]	OC-Cre	Cx43	Mice with Cx43 deficient osteoblasts showed significantly greater anabolic response to mechanical loading.
McBride-Gagyi et al. (2015) [49]	UBC-Cre OSX-Cre Vec-Cre	BMP-2	Endothelial cells and osteoblasts are not a source of BMP-2 for endochondral fracture healing. Non-endochondral fracture healing does not depend on BMP-2.
Phillips et al. (2008) [50]	Colα1-Cre	beta1 integrin	The absence of mechanical loading typically causes changes to cortical bone geometry. Deletion of Beta1 integrins resulted in fewer changes to cortical geometry proving that Beta1 integrins are involved in mechanotransduction.
Shekaran et al. (2014) [51]	Twist-Cre Osterix-Cre Osteocalcin-Cre	Beta1 integrin	Twist-Cre: Mice had severe skeletal impairment and died at birth. Beta1 is responsible for skeletal ossification. Osterix-Cre: Beta1 deletion impacted incisor eruption and the formation of perinatal bone. Osteocalcin-Cre: Beta 1 deletion had only minor skeletal effects.
Delgado-Calle et al. (2016) [52]	(DMP1)-8kb- expressing cells	Parathyroid hormone receptor (Pth1r)	Pth1r regulates basal bone resorption levels and is required for anabolic actions of mechanical loading.
Iura et al. (2015) [53]	Col1-CreERTM	Bmpr1a	Lower Bmpr1a signaling makes osteoblasts more sensitive to mechanical loading and improves the mechanical properties of bone.
Grimston et al. (2009) [54]	Col-Cre	Gja1	Deletion of Gja1 reduces the anabolic response to mechanical loading.
Lawson et al. (2021) [55]	Osx-CreERT2	Wnt1 and Wnt7b	Wnt ligands are required to maintain homeostasis in adult bones and control the anabolic response to mechanical loading.
Mahon et al. (2015) [56]	Col1α2-Cre	(miR)17–92	The periosteal bone response to mechanical strain is reduced without (miR)17–92. (miR) 17–92 plays a role in regulating type 1 collagen during periosteal bone formation.
Lau et al. (2015) [57]	DMP1-Cre	Igf1	Igf1 is required for the anabolic response to mechanical loading, but it is not required for bone repletion.
Lau et al. (2013) [58]	DMP1-Cre	Igf1	Deletion of Igf1 prevents the activation of Wnt signaling in response to a mechanical load. Igf1 impacts the mechanosensitivity of bone.
Temiyasathit et al. (2012) [59]	Colα(1)2.3-Cre	Kif3a	Deletion of Kifa3 leads to decreased bone formation suggesting that primary cilia are mechanosensors for bone.
Grimston et al. (2012) [60]	DM1-Cre	Gja1	Deletion of Gja1 results in Cx43 deficiency and increases the periosteal and endocortical responses of bone to axial compression.
Zhao et al. (2013) [61]	Dmp-Cre	Lrp5	Deletion of Lrp5 decreases mechanoresponsiveness and bone mass, and increases elasticity.
Kesavan et al. (2011) [62]	Col1α2-Cre	Igf1	Igf1 is required for the transduction of a mechanical signal into a signal for the anabolism of bone.
Xiao et al. (2011) [63]	Dmp1-Cre	Pkd1	Pkd1 is required to initiate the anabolic response to mechanical loading of osteoblasts and osteocytes.
Castillo et al. (2012) [64]	FAK^−/−^ clone ID8	FAK	FAK is required for mechanical signaling in vitro but not in vivo.
